# Online-dominant self-management is associated with lower, while hybrid mode is associated with higher, quality of life: a community-based cross-sectional study

**DOI:** 10.3389/fpubh.2026.1911349

**Published:** 2026-09-03

**Authors:** Xuanyu Wang, Fan He, Qinli Zhang, Yang Meng, Ting Chen, Ye Zhu

**Affiliations:** 1 Shanghai Baoshan District Center for Disease Control and Prevention (Shanghai Baoshan District Health Inspection and Supervision Institute), Shanghai, China; 2 Shanghai Baoshan Patriotic Health and Health Promotion Guidance Center, Shanghai, China

**Keywords:** community-based health promotion, digital divide, health-related quality of life, middle-aged and older adults, online-dominant self-management, online-offline integrated mode, self-management

## Abstract

**Background:**

Health-related quality of life (HRQoL) is an important outcome measure for evaluating community health interventions. Exploring effective community-based health self-management strategies is of practical significance in the context of population aging. This study compared the associations of different activity delivery modes (hybrid online-offline, offline-dominant, and online-dominant) implemented within community health self-management groups on residents’ HRQoL, and explored associations with activity-related factors and demographic factors.

**Methods:**

A cross-sectional survey was conducted among 1,697 residents in Baoshan District, Shanghai. The 36-Item Short Form Health Survey (SF-36) was used to assess Physical Component Summary (PCS) and Mental Component Summary (MCS) scores. Multiple linear regression was employed to adjust for demographic covariates, and estimated marginal means were used to estimate between-group mean differences (MD) with 95% confidence intervals (CI).

**Results:**

After adjusting for demographic factors, the hybrid online-offline group had significantly higher PCS (MD = 3.96, 95% CI: 1.65 to 6.26) and MCS (MD = 5.44, 95% CI: 3.10 to 7.79) compared with general residents (both *p* < 0.001). In contrast, the online-dominant group had significantly lower PCS (MD = −6.84, 95% CI: −10.01 to −3.67, *p* < 0.001) and MCS (MD = −5.72, 95% CI: −8.98 to −2.46, *p* < 0.01). The offline-dominant group showed no significant differences from general residents. The exercise-only group had significantly higher MCS than general residents (MD = 5.60, 95% CI: 1.18 to 10.04, *p* < 0.05). Activity frequency of no more than once per week was associated with higher PCS (MD = 3.20, 95% CI: 0.96 to 5.45, *p* < 0.01) and MCS (MD = 3.14, 95% CI: 0.86 to 5.43, *p* < 0.01), whereas frequency exceeding twice per week was associated with lower MCS (MD = −4.11, 95% CI: −7.19 to −1.04, *p* < 0.01). Female sex, older age, and poorer economic status were associated with lower PCS.

**Conclusion:**

In this community-based cross-sectional study, the hybrid online-offline mode was associated with better HRQoL, while the online-dominant mode was associated with poorer HRQoL. Exercise activities were associated with better mental health; however, higher frequency was not necessarily associated with better outcomes. Participation at a frequency of no more than once per week was associated with higher HRQoL scores. Given the cross-sectional design, these findings should be interpreted as associative and hypothesis-generating rather than causal, and prospective studies are needed to confirm the observed associations.

## 1 Introduction

Currently, the global population is experiencing accelerated aging, with estimates suggesting that individuals aged 65 and above will account for 20% of the world‘s population by 2050 (1). In many countries, this proportion has already exceeded this threshold, and some countries have already peaked in population size by 2024, creating an urgent need for community health promotion (2). In this context, health-related quality of life (HRQoL), a multidimensional indicator encompassing physical, psychological, and social functioning, has been widely used to assess population health and evaluate the effectiveness of public health interventions (3). The present study focuses on middle-aged and older adults, broadly defined as individuals aged 45 years and older, which is an age range that covers both middle and older adulthood, consistent with prior studies that have used ≥50 years as the cutoff for older adults or defined middle-aged adults as those aged 45–60 or 45–64 years (4–7). Against this backdrop, identifying sustainable, high-coverage community-based health promotion strategies to maintain and improve HRQoL among this population has become a public health priority of urgent global significance (8).

The Resident Health Self-Management Group is a community-based participatory health promotion model grounded in the core principles of patient-provider collaboration, mutual support among residents, and self-management. Previous studies have mostly focused on patients with chronic diseases, confirming that participation in such groups can improve disease-related outcomes and quality of life (9–11). For general community residents, who may not have diagnosed conditions but nevertheless face age-related declines in physical activity, musculoskeletal function, psychological well-being, and social support, as well as the accumulation of multimorbidity, the potential benefits of such groups are equally relevant (12). In this population, health self-management interventions carry dual significance: for healthy or sub-healthy residents, they may help maintain current health status and delay functional decline; for those with existing conditions, they can promote disease self-management, symptom control, and overall quality of life. Despite these potential benefits, however, empirical evidence on the effectiveness of health self-management groups among general community-dwelling populations remains limited.

The COVID-19 pandemic has greatly accelerated the adoption and application of digital health technologies, reshaping the delivery of community-based health interventions. While these technologies are believed to reduce healthcare costs, improve accessibility, enhance care quality, and facilitate personalized treatment, their effectiveness among populations with low digital literacy (such as middle-aged and older adults, and those with lower socioeconomic status) remains controversial, with some studies raising concerns about exacerbating the digital divide and health inequalities (4, 13–15). In this context, hybrid models that integrate online and offline approaches have emerged as a promising alternative, and have demonstrated potential for improving HRQoL among patients with chronic diseases (16–18). However, systematic evidence regarding the effectiveness and optimal activity models of hybrid interventions in health self-management groups for general, non-clinical community populations remains limited.

The present study was conducted in Baoshan District, Shanghai, where a mature network of over 400 health self-management groups has been established since 2009, providing a well-developed platform to examine these questions. Accordingly, we targeted both health self-management group members and general residents in this district, with three main objectives corresponding to the analyses described above: (1) to compare the associations of different activity delivery modes (hybrid online-offline, offline-dominant, and online-dominant) with HRQoL, using general residents as the reference group; (2) to examine how different activity contents (exercise-only, non-exercise-only, and exercise combined with other activities) and activity frequencies are associated with HRQoL; and (3) to identify demographic factors independently associated with HRQoL among group members. We hypothesized that: (1) members of the hybrid online-offline group would report higher HRQoL than general residents, whereas the online-dominant group would report lower HRQoL; (2) participating in exercise activities would be associated with higher HRQoL; (3) the relationship between activity frequency and HRQoL would be non-linear, with moderate frequency showing favorable associations relative to very low or very high frequency; and (4) female sex, older age, and lower economic status would be associated with poorer HRQoL. By addressing these objectives, this study aims to fill the aforementioned research gaps and provide empirical evidence for optimizing community-based health promotion strategies in aging societies. Given that many countries facing severe population aging—such as Japan, Germany, and Italy—encounter similar challenges in chronic disease management, health service delivery, and digital equity, the evidence generated from this well-established community practice in China may offer valuable references for the design of integrated online-offline health promotion strategies in comparable international contexts (2).

## 2 Methods

### 2.1 Data resource

Data were collected between March and April 2024. A stratified random cluster sampling method was employed.

#### 2.1.1 Health self-management group definition

The resident health self-management group is a community-based voluntary organization initiated by the Patriotic Health Campaign Committee, led by subdistrict/town governments, organized by neighborhood/village committees, and guided by health professionals. It operates on the core principles of patient-provider collaboration, mutual support among residents, and self-management. Through diverse interactive activities including peer education, health knowledge sharing, and exercise activities, the model aims to equip residents with scientific health knowledge and skills, improve health literacy, and enable them to appropriately apply self-management competencies to gain control over health-related decisions and actions. Each group typically consists of 15–20 members, led by a volunteer group leader and supported by a community physician or trained health professional.

#### 2.1.2 Sampling and recruitment

The health self-management groups were first stratified into three temporal strata according to their year of establishment: (1) ≤ 2012, (2) 2013–2017, and (3) 2018–2022. From each stratum, one group was randomly selected from each of the 17 community health centers in Baoshan District, yielding 3 selected groups per center and 51 groups in total. To reduce selection bias, all members of the selected groups (≥15 members per group) were invited to participate. Meanwhile, general residents who had never participated in any health self-management group activities were recruited from the same neighborhood committees, with assistance from committee directors, aiming for a roughly equal number with comparable age range and sex ratio to the group members. The survey was jointly initiated by the Baoshan District Center for Disease Control and Prevention and the Baoshan District Patriotic Health and Health Promotion Guidance Center. A formal notification was issued to all subdistrict/town health offices to coordinate the survey. Trained investigators administered the electronic questionnaire face-to-face at community centers, and participants were assured of confidentiality and anonymity to encourage truthful reporting, which minimized social desirability bias.

Inclusion criteria: (1) age ≥18 years; (2) residency in Baoshan District for at least 6 months; (3) ability to understand and complete the questionnaire independently or with assistance from trained investigators. Exclusion criteria: (1) severe cognitive impairment or psychiatric disorders that prevented completion of the questionnaire; (2) severe physical illness or communication barriers that precluded participation.

#### 2.1.3 Sample size calculation

The sample size was estimated using the formula for comparing two independent means:


n=2(Zα2+Zβ)2σ2δ2×Deff×11−r

In this formula, Zα2 =1.96 (two-sided significance level α =0.05), and $Z_{\beta }$ =0.84 (statistical power of 80%). σ=10.18 , which was the larger standard deviation of the Physical Component Summary (PCS) and Mental Component Summary (MCS) scores of the 36-Item Short Form Health Survey (SF-36) from a previous community-based study among Chinese middle-aged and older adults (19). Taking the minimal clinically important difference (MCID) of 2.5 points for both PCS and MCS to be the detectable difference (i.e., δ=2.5 ) (20). Considering the stratified cluster sampling design, a design effect (Deff) of 2.0 was applied. An anticipated non-response rate of 20% was also included (i.e., r = 0.2). Substituting these values yielded a required sample size of approximately 650 participants per group, or 1,300 in total for group members and general residents. In practice, we surveyed all members of the selected groups and a roughly equal number of general residents from the same communities. Of the 1,703 questionnaires returned, 6 were excluded due to garbled age data, yielding a final analytic sample of 1,697 valid cases (exclusion rate: 0.35%). All subsequent statistical analyses, including descriptive statistics, between-group comparisons, and regression models, were performed on this complete dataset, thus no missing data imputation was required. The final sample exceeded the minimum required sample size, providing adequate statistical power for the main analyses.

### 2.2 Survey methods

A self-designed questionnaire was administered in electronic form. The first section collected data on general demographic characteristics and health self-management-related factors. The categorization framework for activity-related variables was developed through literature review and local practice standards, and was independently reviewed by three community health promotion experts to ensure content validity. A pilot test was conducted in one community (*n* = 31) prior to the formal survey to evaluate face validity, and 93.55% (29/31) of respondents considered the category options clear and accurate. In addition, cross-checking the 31 pilot responses against available group activity logs yielded an agreement rate of 91.94%, providing preliminary evidence for classification reliability. Based on pilot feedback, the wording of several questions and the response options for activity-related items were refined to enhance comprehension among middle-aged and older participants. The second section employed the 36-Item Short Form Health Survey (SF-36) to assess HRQoL, and the Physical Component Summary (PCS) and Mental Component Summary (MCS) scores were calculated to reflect the overall physical and mental health status. Higher scores indicate better health status. The SF-36 is a commonly used tool for assessing HRQoL in older adults (8). The SF-36 has been extensively validated in general populations, with previous studies reporting excellent internal consistency (Cronbach’s *α* > 0.90 for both PCS and MCS) and good construct validity (8, 21). Additionally, for group members, the following variables were assessed using the self-designed questionnaire: activity delivery mode (offline-dominant, online-dominant, or hybrid online-offline), activity content (exercise-only, non-exercise-only, or exercise + other activities), and activity frequency (≤1 time/month, ≤1 time/week, or ≥2 times/week). Online activities were defined as those conducted via digital platforms (e.g., live-streamed lectures, online exercise, or health information sharing via social media); offline activities referred to in-person sessions (e.g., Baduanjin, Tai Chi, or face-to-face lectures). Hybrid mode was defined as a roughly balanced frequency of both online and offline participation. Activity content was categorized as: (1) exercise-only (e.g., aerobic exercises including Baduanjin, Tai Chi, outdoor walking), (2) non-exercise-only (e.g., health lectures, knowledge sharing, health assessments, or health plan development), or (3) exercise combined with other activities (participation in both types). To minimize information bias, all investigators provided standardized examples for these classifications during the face-to-face interviews, and activity-related questions were anchored to the past 12 months to reduce recall bias. For group members, responses were cross-referenced with group activity logs where available. All interviewers received standardized training, and onsite quality controllers reviewed completed questionnaires for completeness and logic.

### 2.3 Statistical analysis

Data were entered using EpiData and analyzed using Stata 15.1 software. Continuous variables are presented as mean ± standard deviation (X ± S), and categorical variables as frequencies (%). Differences in basic characteristics between health self-management group members and general residents were compared using independent samples t-test for continuous variables and chi-square test for categorical variables. Univariate analysis of factors associated with PCS and MCS were performed using independent samples t-test and one-way ANOVA as appropriate. Normality of continuous variables was assessed using Shapiro–Wilk tests, and homogeneity of variance was evaluated using Levene’s tests. To compare HRQoL between group members with different activity delivery modes, activity contents, activity frequencies, and general residents, we used multiple linear regression models that included age, sex, education level, living arrangement, economic status, and retirement status as demographic covariates. Estimated marginal means and between-group mean differences (MD) with 95% confidence intervals (95% CI) were calculated. The effect size was expressed as Cohen’s d. Forest plots were generated using R 4.5.2 software to visually present the effects of different activity delivery modes on PCS and MCS. Line charts were plotted using Microsoft Excel to visually display the relationship between activity frequency and PCS and MCS. Activity frequency was set as the horizontal axis, and the adjusted means of PCS and MCS as the vertical axis, with the levels of general residents serving as dashed reference lines. Error bars represent 95% confidence intervals. Multiple linear regression analysis was further employed to identify factors independently influencing PCS and MCS among group members. Variables included in the model were selected based on two criteria: (1) variables that showed significant associations with PCS or MCS in univariate analysis; and (2) established demographic determinants of HRQoL from the literature, including age and sex, which were retained in the multivariable models regardless of their univariate significance levels to ensure comparability with previous studies and to control for potential confounding. Variance inflation factor (VIF) was calculated to assess multicollinearity. The significance level was set at *α* = 0.05.

## 3 Results

### 3.1 Basic characteristics

A total of 1,703 questionnaires were returned, of which 1,697 were valid and retained for analysis, corresponding to a valid response rate of 99.6%. The study sample comprised 892 health self-management group members (52.56%) and 805 general residents (47.44%), with a mean age of 57.54 ± 14.09 years. There were significant differences between group members and general residents in age, gender, education level, and retirement status (all *p* < 0.05). Specifically, compared with general residents, group members had a higher proportion of females, were more likely to be retired, and had lower educational attainment. See Table 1. Since age was a continuous variable, t-test results for age demonstrated that group members were on average 5.35 years older (60.08 ± 12.91 vs. 54.73 ± 14.80 years, t = 7.955) than general residents.

**Table 1:** Basic characteristics of members of health self-management groups and general residents (*N* = 1,697).

Variable	Indicators	Total (*N* = 1,697)	Group members (*n* = 892)	General residents (*n* = 805)	χ^2^	*p*
		*n* (%)	*n* (%)	*n* (%)		
Gender	Male	577 (34.00)	260 (29.15)	317 (39.38)	χ^2^ = 19.737	<0.001^***^
	Female	1,120 (66.00)	632 (70.85)	488 (60.62)		
Education level	Junior high school or below	691 (40.72)	397 (44.51)	294 (36.52)	χ^2^ = 22.568	<0.001^***^
	Senior high school/ technical secondary school	450 (26.52)	248 (27.80)	202 (25.09)		
	Junior college or above	556 (32.76)	247 (27.69)	309 (38.39)		
Living arrangement	Living alone	125 (7.37)	63 (7.06)	62 (7.70)	χ^2^ = 2.380	0.497
	Living with spouse/partner	808 (47.61)	435 (48.77)	373 (46.34)		
	Living with children (two generations)	513 (30.23)	272 (30.49)	241 (29.94)		
	Living with three or more generations	251 (14.79)	122 (13.68)	129 (16.02)		
Economic status	Satisfied	715 (42.13)	385 (43.16)	330 (40.99)	χ^2^ = 1.245	0.537
	Fair	911 (53.68)	473 (53.03)	438 (54.41)		
	Dissatisfied	71 (4.18)	34 (3.81)	37 (4.60)		
Retirement status	Yes	1,081 (63.70)	648 (72.65)	433 (53.79)	χ^2^ = 65.070	<0.001^***^
	No	616 (36.30)	244 (27.35)	372 (46.21)		

^*^p < 0.05; ^**^p < 0.01; ^***^p < 0.001.

### 3.2 Univariate associations of demographic factors with HRQoL

Univariate analyses across the full sample (Supplementary Table 1, *N* = 1,697) identified education levels and economic status as factors significantly associated with PCS (both *p* < 0.001). MCS scores differed significantly across education levels (*p* < 0.001), living arrangements (*p* < 0.05), economic status groups (*p* < 0.001), and retirement status (*p* < 0.01). Specifically, participants who were retired had 2.98 points higher MCS than those who were not retired.

### 3.3 Associations of activity-related factors with HRQoL

**Figure 1:**
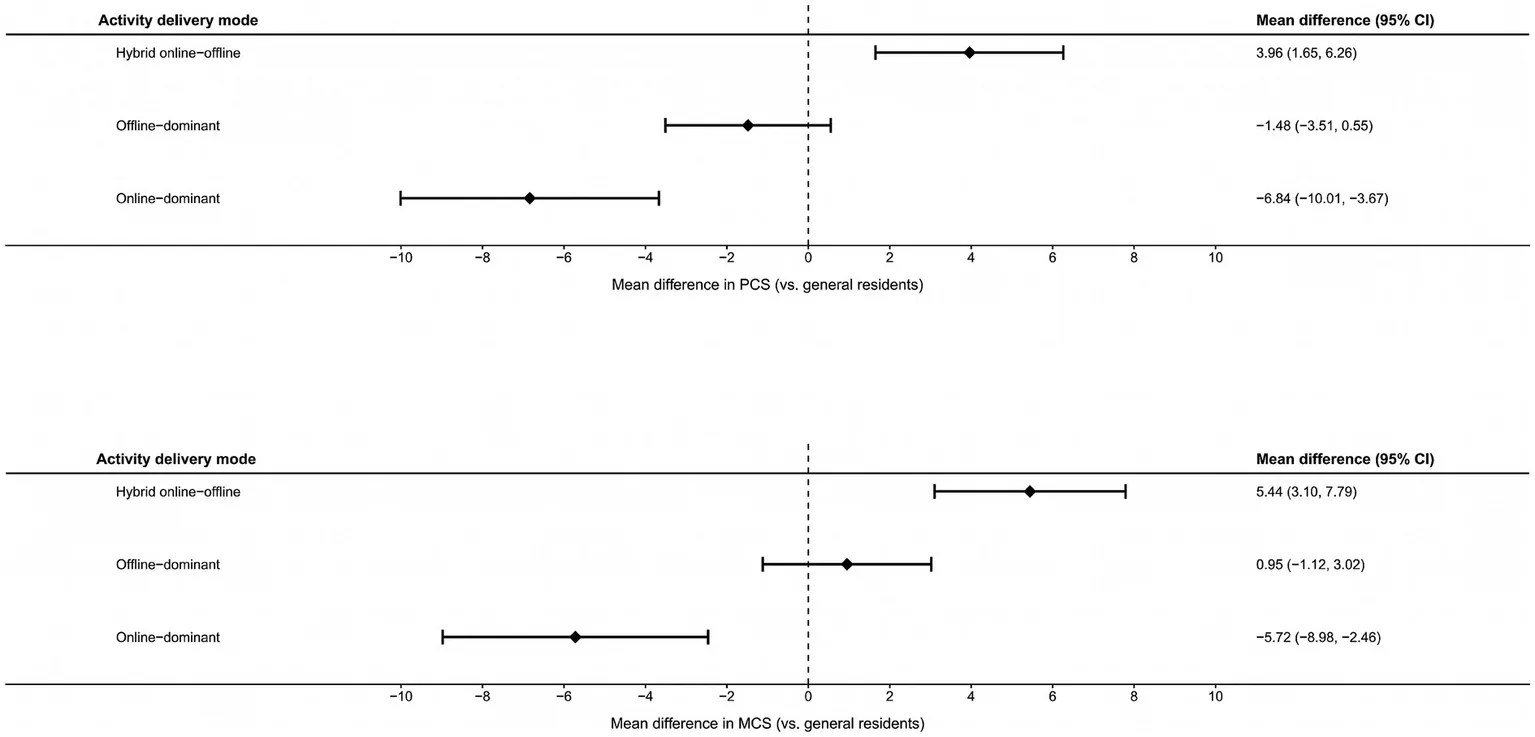
Forest plot of the associations of activity delivery modes with PCS and MCS scores.

We included 892 group members and 805 general residents (as the reference group) in the adjusted models. After adjustment for demographic covariates, distinct patterns emerged across the three activity delivery modes (Table 2; Figure 1). The hybrid online-offline mode was the only format consistently associated with significantly higher scores on both dimensions relative to non-participation (PCS: MD = 3.96; MCS: MD = 5.44. Both *p* < 0.001). Conversely, the online-dominant mode was associated with significantly lower scores on both dimensions (PCS: MD = −6.84, *p* < 0.001; MCS: MD = −5.72, *p* < 0.01). The offline-dominant mode showed no significant differences from general residents on either dimension. These divergent patterns suggest that it is the integration of online and offline elements, rather than the mere presence of either modality alone, that may characterize the hybrid mode’s favorable association. Figure 1 visually presents these results. The figure shows that the mean difference and confidence interval for the hybrid online-offline group lie entirely to the right of zero (positive association), while those for the online-dominant group lie entirely to the left (negative association), and those for the offline-dominant group cross the zero line (no association).

**Table 2:** Comparison of SF-36 scores between members with different activity delivery modes and general residents (*N* = 1,697).

Sf-36	Group	Adjusted mean (95% CI)	Mean difference (95% CI)	Cohen’s d	*p*
PCS	General residents ^a^	79.56 (78.35,80.78)	-	-	-
	Offline-dominant	78.04 (76.43,79.64)	−1.48 (−3.51,0.55)	−0.10	0.153
	Online-dominant	72.67 (67.73,75.62)	−6.84 (−10.01,-3.67)	−0.10	<0.001^***^
	Hybrid online-offline	83.47 (81.53,85.41)	3.96 (1.65,6.26)	0.23	<0.001^***^
MCS	General residents ^a^	76.59 (75.35,77.83)	-	-	-
	Offline-dominant	77.53 (75.89,79.17)	0.95 (−1.12,3.02)	0.05	0.370
	Online-dominant	70.87 (67.85,73.88)	−5.72 (−8.98,-2.46)	−0.32	0.001^**^
	Hybrid online-offline	82.03 (80.05,84.01)	5.44 (3.10,7.79)	0.31	<0.001^***^

^*^p < 0.05; ^**^p < 0.01; ^***^p < 0.001; ^a^Reference group.

Regarding activity content (Supplementary Table 2), exercise-only participation was associated with higher MCS (MD = 5.60, *p* < 0.05), and exercise combined with other activities also showed a positive MCS association (MD = 3.55, *p* < 0.01). In contrast, non-exercise-only activities were associated with lower PCS (MD = −3.06, *p* < 0.05). This dichotomy suggests that exercise may be more closely associated with mental health than non-exercise activities in this community setting.

**Figure 2:**
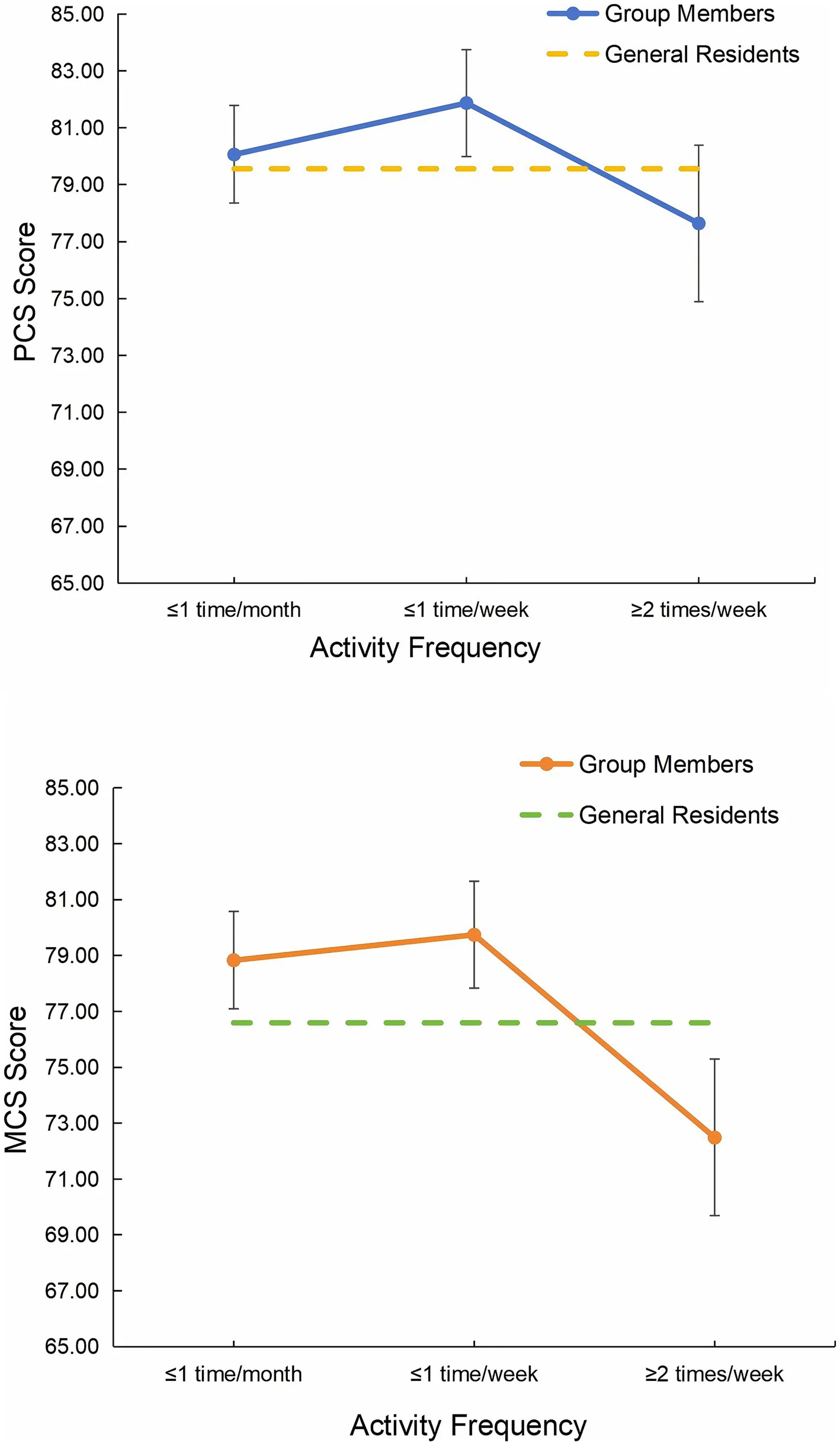
Associations between activity frequency and HRQoL scores.

Notably, the association between activity frequency and HRQoL was non-linear (Supplementary Table 2; Figure 2). Participation at a frequency of once per week or less was associated with both higher PCS (MD = 3.20, *p* < 0.01) and MCS (MD = 3.14, *p* < 0.01). However, frequencies exceeding twice per week were associated with lower MCS (MD = −4.11, *p* < 0.01). Figure 2 further illustrates the nonlinear relationship between activity frequency and PCS (Figure 2A) and MCS (Figure 2B) in the form of line charts. The inverse pattern at higher frequencies suggests that more frequent attendance does not necessarily associate with additional benefit.

### 3.4 Multiple linear regression analysis of factors associated with HRQoL among group members

In the adjusted model restricted to group members (Table 3, *N* = 892), the hybrid mode remained the strongest independent positive predictor for both PCS (*β* = 0.141, *p* < 0.001) and MCS (*β* = 0.138, *p* < 0.001). The online-dominant mode was negatively associated with both PCS (*β* = −0.071, *p* < 0.05) and MCS (*β* = −0.098, *p* < 0.01). Non-exercise-only activities were strongly associated with lower MCS (*β* = −0.233, *p* < 0.001), and frequency ≥2 times/week remained a negative predictor for MCS (*β* = −0.122, *p* < 0.01). After accounting for activity-related factors, several demographic variables that were significant in univariate analyses (including education, living arrangement, and retirement status) no longer reached statistical significance. It suggests that their apparent associations with HRQoL may be largely accounted for by differences in activity participation patterns rather than by direct demographic effects. In contrast, female sex (*β* = −0.097, *p* < 0.05), older age (*β* = −0.079, *p* < 0.05), and poorer economic status remained independently associated with lower PCS, with a clear socioeconomic gradient (fair vs. satisfied: *β* = −0.118, *p* < 0.01; dissatisfied vs. satisfied: *β* = −0.073, *p* < 0.05). These persistent demographic disadvantages, even within a structured group intervention, indicate that such subgroups may require targeted supplementary support. All VIF values in both models were below 4.11, indicating no substantial multicollinearity among predictors.

**Table 3:** Multiple linear regression analysis of factors associated with SF-36 scores among group members (*N* = 892).

Dependent variable	Independent variable (vs. reference group)	*β* (95% CI)	*B* (95% CI)	SE	*t*	*p*
PCS	Hybrid online-offline (vs. offline-dominant)	0.141 (0.071, 0.210)	4.979 (2.505, 7.453)	1.261	3.95	<0.001^***^
	Online-dominant (vs. offline-dominant)	−0.071 (−0.141, −0.001)	−3.371 (−6.688, −0.055)	1.690	−1.99	0.046^*^
	Non-exercise-only (vs. exercise-only)	−0.153 (−0.282, −0.026)	−5.235 (−9.600, −0.870)	2.224	−2.35	0.019^*^
	Female (vs. male)	−0.097 (−0.186, −0.009)	−0.127 (−0.242, −0.011)	0.059	−2.15	0.032^*^
	Age	−0.079 (−0.144, −0.014)	−2.929 (−5.334, −0.525)	1.225	−2.39	0.017^*^
	Fair economic status (vs. satisfied)	−0.118 (−0.184, −0.051)	−3.963 (−6.207, −1.719)	1.143	−3.47	0.001^**^
	Dissatisfied with economic status (vs. satisfied)	−0.073 (−0.140, −0.005)	−6.392 (−12.31, −0.475)	3.015	−2.12	0.034^*^
MCS	Hybrid online-offline (vs. offline-dominant)	0.138 (0.068, 0.207)	4.999 (2.490, 7.508)	1.278	3.91	<0.001^***^
	Online-dominant (vs. offline-dominant)	−0.098 (−0.133, −0.029)	−4.753 (−8.085, −1.422)	1.697	−2.80	0.005^**^
	Non-exercise-only (vs. exercise-only)	−0.233 (−0.359. -0.106)	−8.148 (−12.57, −3.728)	2.252	−3.62	<0.001^***^
	Activity frequency ≥2 times/week (vs. ≤1 time/month)	−0.122 (−0.191, −0.053)	−5.555 (−8.679, −2.431)	1.591	−3.49	0.001^**^

^*^p < 0.05; ^**^p < 0.01; ^***^p < 0.001. Models were adjusted for age, sex, education level, economic status, living arrangement, and retirement status. Full regression results are presented in the Supplementary Table 3.

## 4 Discussion

Health self-management interventions have been shown to be associated with improved HRQoL in chronic disease prevention and control contexts (9). By comparing the associations of different activity models of health self-management groups with residents’ physical and mental dimensions of HRQoL, this study aimed to explore potential community self-management support strategies for the general, non-clinical community population. This study found that the hybrid online-offline mode was associated with higher scores in both physical and mental dimensions of HRQoL. In contrast, the offline-dominant group showed no significant difference in HRQoL compared with general residents, while the online-dominant group had significantly lower scores. To our knowledge, this is the first study to demonstrate these differential associations of activity delivery modes in the context of community health self-management groups, filling an important research gap. In the following discussion, we draw on prior literature to offer potential interpretations of the observed associations. These mechanistic explanations are proposed as testable hypotheses to inform future research, rather than as conclusions established by the present study. Causal interpretations are precluded by the cross-sectional design, and we explicitly note this limitation in the Limitations section.

Many other studies in different patient populations have also reported benefits of hybrid online-offline modes (16–18). Although these studies focused on patient populations, their proposed mechanisms may offer useful insights for understanding the observed associations in community-dwelling populations. A quasi-Experimental study implementing a hybrid intervention for hemodialysis patients found that this model significantly improved patients’ disease knowledge and self-management ability (16). It was demonstrated that the underlying mechanisms may be that online activities provide standardized, repeatable health knowledge, while offline activities address individualized problems through face-to-face follow-up, consultation, and guidance, translating knowledge into actual health behaviors (16–18). In addition, researchers have found that, from the perspective of tie strength, strong ties obtained from offline activities (such as family members, close friends, and community doctors with long-term face-to-face interaction) provide stable emotional belonging and practical support, while weak ties from online activity groups (e.g., lecture groups) provide accessible information and low-burden external motivation (22–24). This combination has been proposed to enhance participants’ psychological resilience, enabling them to face health problems more positively, and to reduce psychological resistance and anxiety, thereby facilitating the transition to health empowerment (active learning, self-monitoring, adherence to exercise, etc.) and long-term maintenance of health behaviors (25). This has been conceptualized as a chain pathway of “psychological resilience - health empowerment - health behavior maintenance” (26). This offers a potential mechanistic explanation for the observed associations of the hybrid mode with HRQoL, from the perspective of coordinated psychological adaptation and physiological functioning (27). The offline-dominant mode lacks the information expansion provided by weak ties, while the online-dominant mode lacks the emotional anchoring of strong ties. Therefore, neither can fully realize this proposed chain. However, evidence is not uniformly positive, a systematic review of complex community interventions for older adults found that while such interventions reduced mortality, enhanced cognitive function, they did not significantly improve quality of life (28). The discrepancy may reflect differences in study design: RCTs test causal effects while cross-section studies offer associational evidence. The difference in target populations may also be the reason, as Ho et al. targeted older frail adults, while the participants in our study were community-dwelling middle-aged and older adults, who might have better health profiles. These distinctions suggest that the hybrid mode’s observed advantages in our study, while not directly comparable to RCT evidence, may represent a complementary real-world perspective on activity delivery formats within existing community platforms.

Turning to the disadvantages of the online-dominant mode, this study found that HRQoL in the online-dominant group was even significantly lower than that of general residents. A possible interpretation of this finding is the digital divide, which has been shown by other study to be significantly negatively associated with HRQoL (29). The digital divide refers to disparities across populations in accessing and utilizing information and communication technologies (30). Digital literacy is its determinant core dimension, primarily manifested as differences in accessibility of digital resources, proficiency in digital skills, and participation in digital activities (30). However, it should be noted that digital literacy was not directly measured in the present study, the following mechanistic interpretations remain hypothetical rather than empirically demonstrated. Although some reviews indicate that, overall, middle-aged and older adults perceive positive effects of using digital health interventions on their behavior change, disease acceptance, etc., and report improvements not only in disease-related indicators but also in mental health awareness, knowledge, and skills (4, 31, 32). These studies also clearly note that the benefits are highly dependent on the mode of use, content nature, and population characteristics (4, 31, 32). Active participation and emotional support may contribute to social well-being, whereas negative content and adverse social comparisons may exacerbate psychological burden (32). In most studies, middle-aged and older adults report varying levels of digital literacy, and those familiar with digital technology show higher confidence and comfort in using online interventions, whereas low digital literacy has been identified as a barrier (4). The population of this study was predominantly middle-aged and older adults (mean age 57.5 years) and female, with nearly half having education below senior high school level and over 50% reporting fair or poor economic status. These characteristics have been associated with lower digital health literacy (15, 33). Thus, one could hypothesize that these community residents might not only face technological accessibility issues in online activities, but could also experience problems such as overload of low-quality information, digital anxiety, and social isolation, which may impair mental health (15, 30, 34). Besides the potential impact of digital literacy, interpersonal interaction influence may also play an important role in middle-aged and older adults’ participation in online health interventions. Previous research suggests that although online platforms provide the convenience of remote contact with physicians, they tend to supplement online activities with face-to-face communication (4). Similarly, a systematic review by Mills et al. (32) pointed out that while online support groups can provide emotional and informational support, they may not replace the stable emotional anchoring and behavioral supervision brought by offline strong ties, further suggesting the limitations of the online-dominant mode in the context of community-based health self-management among middle-aged and older adults.

Notably, the clinical and public health relevance of these findings deserves further consideration, particularly when interpreted through the lens of the Cohen’s d effect sizes. Although the Cohen‘s d values for the between-group differences (hybrid mode group vs. General residents: 0.23 for PCS and 0.31 for MCS; online-dominant group vs. General residents: −0.10 for PCS and −0.32 for MCS) only reach small effects by conventional benchmarks, the clinical and public health relevance of our findings warrants attention. First, the observed mean differences between the hybrid group and general residents exceeded the established minimal clinically important differences (MCID) for the SF-36 (≥2.5 for PCS and MCS in general populations), indicating that the associations are clinically meaningful (20). However, as a community-based strategy (not a clinical one), the hybrid mode has limited direct effects on physiological outcomes and does not replace pharmacological or surgical interventions. Second, in the context of community-based public health interventions, if the intervention can be implemented broadly, even small individual-level effect sizes can translate into substantial population-level health gains (35, 36). The health self-management group program has been running in Baoshan District for 17 years, over 400 health self-management groups have been established, and a total of 50,200 resident attendances have been recorded in the group activities to date. Benefiting from a high-quality resource integration mechanism that coordinates social work and health departments, along with established community facilities and digital platforms, this well-developed organizational network enables the hybrid mode to be scaled at relatively low cost without compromising broad population reach. Therefore, even modest individual-level effect sizes may yield meaningful cumulative health benefits when implemented through such an established, scalable community platform.

This study also examined the associations of exercise and activity frequency with HRQoL. The results showed that participation in non-exercise activities only (e.g., lectures) was associated with lower PCS and MCS scores. In contrast, those who engaged in exercise activities (whether exercise-only or exercise combined with other activities) had significantly higher MCS than general residents. These findings are consistent with the notion that exercise may play a particularly important role in relation to mental health (5). From a physiological perspective, regular physical activity has been shown to be associated with improved cardiorespiratory fitness, as well as with the secretion of neurotransmitters such as endorphins and serotonin, and the release of brain-derived neurotrophic factor (BDNF), which in turn support emotional regulation and neuroplasticity (5, 37). From a psychosocial perspective, exercise may alleviate symptoms of depression and anxiety (38). Regular exercise has been shown to provide older adults with social support and a sense of control over their daily routines, which have been linked to better mental health (39, 40). In contrast, passive knowledge-transfer activities (e.g., lectures) alone may be inadequate to engage these potential physiological or psychosocial pathways.

Regarding the effect of activity frequency, this study found that higher frequency was not necessarily associated with better outcomes, and frequencies exceeding twice per week were associated with lower MCS. In a systematic review by White et al. (41), low-frequency activities have been reported to be easier to maintain, avoiding interruptions due to time pressure or physical fatigue. Moreover, high-frequency activities may come to be perceived as “tasks” rather than “enjoyment,” potentially reducing participants’ intrinsic motivation and increasing psychological burden. In contrast to our findings, some studies have not observed negative associations with high-frequency activities (3–5 times per week or more) (5, 37, 40, 42–44). One possible explanation for this discrepancy lies in differences in population health status: participants in those studies were mostly patients with specific conditions (depression, diabetes, stroke, etc.), where immediate functional improvements (such as blood glucose control) and positive psychological feedback from high-frequency activities may offset the fatigue cost of participation, resulting in net benefits. In contrast, our participants were community-dwelling individuals with relatively high HRQoL (mean MCS = 78.44, mean PCS = 80.12) compared with those in other studies (38). This difference suggests that the association between activity frequency and HRQoL may depend on population health status to some extent. Nevertheless, due to the cross-sectional nature of our data, reverse causation cannot be excluded. Individuals with poorer health may attend more frequent activities to seek additional support, which could also explain the negative association between high frequency and MCS.

In addition, this study found that female sex, poorer economic status, and older age were independently associated with lower PCS. Previous studies have reported that women bear a greater burden of non-communicable diseases (e.g., cardiovascular disease, depression, musculoskeletal dysfunction) in old age, which may contribute to lower physical HRQoL compared with men (45, 46). This disparity has been attributed not only to biological differences such as hormonal fluctuations and disease presentation but also to the long-standing neglect of older women in clinical research (45). Notably, the negative associations of digital use may be more pronounced in women, and insufficient offline support may further compound their health disadvantages (34). People with low socioeconomic status often have limited access to resources and generally low health literacy and digital literacy, which may hinder the translation of health information into actual health behaviors and limit improvements in self-efficacy (15, 26). Regarding age, apart from natural declines in physiological function, the accompanying reduction in digital literacy may make it even more difficult for older adults to benefit from digital health resources (15, 29). The overlap between populations with HRQoL disadvantages and those with low digital literacy underscores the importance of hybrid online-offline approaches. This also suggests that future intervention designs for health self-management groups should consider differences in sex, age group and resource equity.

The findings of this study offer hypothesis-generating considerations rather than definitive practice recommendations, given the cross-sectional design and modest effect sizes. With this caveat, health self-management groups may consider adopting hybrid online-offline approaches that combine digital health education with in-person behavioral activities, as this format was consistently associated with better HRQoL. Prioritizing structured exercise at a weekly frequency may confer particular mental health benefits, and differentiated support for disadvantaged subgroups (women, older adults, and those with lower socioeconomic status) may help improve intervention equity. These exploratory considerations warrant confirmation in prospective or experimental studies before policy translation. Future research should also examine the optimal balance of online and offline components, as well as the cost-effectiveness and sustainability of hybrid models in diverse community settings.

## 5 Limitations

This study has several limitations. First, the cross-sectional design precludes causal inference, so reverse causation and self-selection bias cannot be excluded. Recent evidence suggests that social support facilitates exercise adherence in older adults, while low physical activity levels, functional decline, and chronic disease burden are strong predictors of sedentary behavior (12). Thus, we may speculate that individuals with better health profiles, higher physical activity levels, or stronger social support may preferentially select hybrid or offline modes, exercise activities and lower activity frequencies, whereas those with poorer health or functional limitations may choose online-only participation, fewer exercise activities, and frequent attendance to seek additional support. This could explain the favorable associations of the hybrid mode, as well as the negative associations between high frequency, non-exercise activities and HRQoL. Second, since chronic disease status, physical function, baseline health status, social support, and physical activity level also influence HRQoL, they can be potential confounders (12, 47, 48). However, they were not measured and thus could not be adjusted for. Their omission, combined with possible reverse causation, may lead to overestimation of the observed associations between activity mode, frequency and HRQoL. Future prospective studies with direct measures of these variables are needed to confirm the observed associations. Current findings should therefore be interpreted as associative and hypothesis-generating rather than causal. Third, the activity-related variables are single-item categorical measures. Their content and face validity were supported by expert review and pilot testing, and reliability was preliminarily supported by cross-verification with activity logs in the pilot sample (*n* = 31, agreement rate = 91.94%). However, this validation was based on a small sample from a single community and did not extend systematically to the full sample. In addition, the classification of activity delivery modes relied on self-report, which may introduce information and recall bias and could exaggerate the observed associations, although investigators provided standardized examples during face-to-face interviews and anchored questions to the past 12 months to minimize such bias. Fourth, our sampling strategy, selecting participants nested within groups and communities, introduces a hierarchical data structure. The current analysis did not account for this clustering effect, which may lead to underestimated standard errors and overestimated statistical significance. Future studies with similar cluster-sampled designs should employ multilevel models to obtain more accurate and generalizable effect estimates. Fifth, the generalizability of our findings remains uncertain. The sample was drawn exclusively from Baoshan District, Shanghai. The target population predominantly consisted of middle-aged and older adults with a high proportion of females and relatively low education level. Generalization of the findings to other regions, younger or older populations should be made with caution. Sixth, although the SF-36 is a widely used and validated instrument, it reflects self-perceived health status rather than objective clinical measures, and cannot distinguish short-term fluctuations from long-term trends in HRQoL. Future prospective cohort or randomized controlled trial designs, combined with digital usage logs and qualitative interviews, are warranted to further validate the long-term effects and applicability of the hybrid model.

## 6 Conclusion

In community-based health self-management groups, the hybrid online-offline mode was associated with better HRQoL compared with non-participation, while the online-dominant mode was associated with poorer HRQoL. Exercise activities showed an association with better mental health, yet higher frequency was not necessarily better. Participation at a frequency of no more than once per week was associated with higher HRQoL scores. Female sex, lower economic status, and older age were identified as risk-associated factors for poorer physical HRQoL. From a practice perspective, health self-management groups may consider hybrid online-offline formats that combine digital knowledge delivery with in-person behavioral activities, and may prioritize structured exercise at a weekly frequency. Differentiated support for disadvantaged groups could also be considered to promote intervention equity. Given the cross-sectional design and potential confounding, the results must be interpreted cautiously, and causal conclusions cannot be drawn. Future longitudinal, multicenter, and interventional studies are recommended to clarify causal relationships and improve generalizability.

## Data Availability

The raw data supporting the conclusions of this article will be made available by the authors, without undue reservation.
