# Supplementary material for: Online-dominant self-management is associated with lower, while hybrid mode is associated with higher, quality of life: a community-based cross-sectional study
Source: Front Public Health. 2026 Sep 3;14:1911349. doi: 10.3389/fpubh.2026.1911349 (PMC13581834; doi:10.3389/fpubh.2026.1911349)
Supplement: Supplementary file 1 [file Table_1.DOCX]

**Supplementary Material**

**Supplementary Table 1.** SF-36 scores of participants by different demographic characteristics (N=1,697)

| **Variable** | **Indicators** | **n (%)** | **PCS** | **MCS** |
| --- | --- | --- | --- | --- |
| Gender | Male | 577 (34.00) | 80.46±18.22 | 77.95±18.30 |
|  | Female | 1120 (66.00) | 78.70±17.32 | 76.92±17.66 |
| t | | | 1.94 | 1.12 |
| *p* | | | 0.052 | 0.262 |
| Education level | Junior high school or below | 691 (40.72) | 77.57±18.02 | 76.42±17.69 |
|  | Senior high school/ technical secondary school | 450 (26.52) | 81.52±17.04 | 80.53±16.89 |
|  | Junior college or above | 556 (32.76) | 79.66±17.46 | 75.68±18.58 |
| F | | | 7.03 | 10.57 |
| *p* | | | <0.001^***^ | <0.001^***^ |
| Living arrangement | Living alone | 125 (7.37) | 76.95±18.37 | 73.49±20.15 |
|  | Living with spouse/partner | 808 (47.61) | 78.85±18.17 | 76.92±18.14 |
|  | Living with children (two generations) | 513 (30.23) | 79.85±16.80 | 77.71±16.68 |
|  | Living with three or more generations | 251 (14.79) | 80.80±17.15 | 79.39±18.00 |
| F | | | 1.69 | 3.26 |
| *p* | | | 0.167 | 0.021^*^ |
| Economic status | Satisfied | 715 (42.13) | 81.89±18.01 | 78.74±19.13 |
|  | Fair | 911 (53.68) | 77.87±16.67 | 76.71±16.46 |
|  | Dissatisfied | 71 (4.18) | 71.55±21.59 | 69.62±20.11 |
| F | | | 17.87 | 9.44 |
| *p* | | | <0.001^***^ | <0.001^***^ |
| Retirement status | Yes | 1081 (63.70) | 78.87±17.88 | 78.35±17.71 |
|  | No | 616 (36.30) | 80.06±17.21 | 75.37±18.04 |
| t | | | -1.34 | 3.31 |
| *p* | | | 0.181 | 0.001^**^ |

^*^*p* < 0.05. ^**^*p* < 0.01. ^***^*p* < 0.001.

**Supplementary Table 2.** Comparison of SF-36 scores between members with different activity content and frequency and general residents

| **Sf-36** | **Activity factor** | **Group** | **Adjusted mean (95% CI)** | **Mean difference**  **(95% CI)** | **Cohen's d** | ***p*** |
| --- | --- | --- | --- | --- | --- | --- |
| PCS | - | General residents ^a^ | 79.56 (78.35,80.78) | - | - | - |
|  | Activity content | Exercise-only | 82.48 (78.32,86.64) | 2.92 (-1.42,7.26) | 0.17 | 0.187 |
|  |  | Non-exercise-only | 76.50 (74.72,78.27) | -3.06 (-5.23,-0.90) | -0.20 | 0.006^**^ |
|  |  | Exercise + other activities | 80.67 (79.05,82.28) | 1.10 (-0.93,3.14) | 0.06 | 0.287 |
|  | Activity frequency | ≤ once/month | 80.06 (78.35,81.78) | 1.40 (-0.72,3.52) | 0.09 | 0.195 |
|  |  | ≤ once/week | 81.87 (79.99,83.74) | 3.20 (0.96,5.45) | 0.21 | 0.005^**^ |
|  |  | ≥ twice/week | 77.64 (74.89,80.39) | -1.02 (-4.04,2.00) | -0.06 | 0.506 |
| MCS | - | General residents ^a^ | 76.59 (75.35,77.83) | - | - | - |
|  | Activity content | Exercise-only | 82.21 (77.96,86.46) | 5.60 (1.18,10.04) | 0.32 | 0.013^*^ |
|  |  | Non-exercise-only | 74.78 (72.97,76.59) | -1.82 (-4.03,0.39) | -0.10 | 0.107 |
|  |  | Exercise + other activities | 80.15 (78.51,81.80) | 3.55 (1.48,5.62) | 0.20 | 0.001^**^ |
|  | Activity frequency | ≤ once/month | 78.83 (77.09,80.57) | 2.23 (0.08,4.39) | 0.13 | 0.042^*^ |
|  |  | ≤ once/week | 79.74 (77.84,81.65) | 3.14 (0.86,5.43) | 0.18 | 0.007^**^ |
|  |  | ≥ twice/week | 72.48 (69.68,75.29) | -4.11 (-7.19,-1.04) | -0.23 | 0.009^**^ |

^*^*p* < 0.05. ^**^*p* < 0.01. ^***^*p* < 0.001.

**Supplementary Table 3.** Multiple linear regression analysis of factors influencing SF-36 scores among group members (N=892)

| **Dependent variable** | **Independent variable**  **(vs. reference group)** | ***β***  ***(95% CI )*** | ***B***  ***(95% CI )*** | **SE** | ***t*** | ***p*** | **VIF** | ***p for model*** |
| --- | --- | --- | --- | --- | --- | --- | --- | --- |
| PCS | Female (vs. male) | -0.097  (-0.186, -0.009) | -0.127  (-0.242, -0.011) | 0.059 | -2.15 | 0.032^*^ | 1.93 | <0.001^***^ |
|  | Age | -0.079  (-0.144, -0.014) | -2.929  (-5.334, -0.525) | 1.225 | -2.39 | 0.017^*^ | 1.04 |  |
|  | Senior high school/technical secondary school (vs. junior high or below) | 0.051  (-0.021, 0.123) | 1.930  (-0.770, 4.630) | 1.376 | 1.40 | 0.161 | 1.27 |  |
|  | Junior college or above (vs. junior high or below) | -0.054  (-0.149, 0.040) | -2.043  (-5.582, 1.496) | 1.803 | -1.13 | 0.258 | 2.18 |  |
|  | Fair economic status  (vs. satisfied) | -0.118 (-0.184, -0.051) | -3.963  (-6.207, -1.719) | 1.143 | -3.47 | 0.001^**^ | 1.09 |  |
|  | Dissatisfied with economic status (vs. satisfied) | -0.073  (-0.140, -0.005) | -6.392  (-12.31, -0.475) | 3.015 | -2.12 | 0.034^*^ | 1.11 |  |
|  | Online-dominant  (vs. offline-dominant) | -0.071  (-0.141, -0.001) | -3.371  (-6.688, -0.055) | 1.690 | -1.99 | 0.046^*^ | 1.20 |  |
|  | Hybrid online-offline  (vs. offline-dominant) | 0.141  (0.071, 0.210) | 4.979  (2.505, 7.453) | 1.261 | 3.95 | <0.001^***^ | 1.20 |  |
|  | Non-exercise-only  (vs. exercise-only) | -0.153 (-0.282, -0.026) | -5.235  (-9.600, -0.870) | 2.224 | -2.35 | 0.019^*^ | 4.02 |  |
|  | Exercise + other activities (vs. exercise-only) | -0.064  (-0.193, 0.065) | -2.143  (-6.488, 2.202) | 2.214 | -0.97 | 0.333 | 4.09 |  |
|  | ≤1 time/week  (vs. ≤1 time/month) | 0.061  (-0.009, 0.131) | 2.112  (-0.309, 4.533) | 1.234 | 1.71 | 0.087 | 1.19 |  |
|  | ≥2 times/week  (vs. ≤1 time/month) | -0.036  (-0.106, 0.033) | -1.618  (-4.696, 1.460) | 1.568 | -1.03 | 0.303 | 1.18 |  |
|  | Constant | - | 93.731 | 4.722 | 19.85 | <0.001 | - |  |
| MCS | Female (vs. male) | -0.065  (-0.237, 0.107) | -2.484  (-4.944, -0.025) | 1.253 | -0.74 | 0.462 | 1.06 | <0.001^***^ |
|  | Age | -0.046  (-0.092, 0.000) | -0.062  (-0.226, 0.103) | 0.084 | -1.98 | 0.050 | 3.81 |  |
|  | Senior high school/technical secondary school  (vs. junior high or below) | 0.067  (-0.003, 0.137) | 2.570  (-0.128, 5.267) | 1.374 | 1.87 | 0.062 | 1.24 |  |
|  | Junior college or above (vs. junior high or below) | 0.014  (-0.078, 0.106) | 0.537  (-3.014, 4.089) | 1.810 | 0.30 | 0.767 | 2.14 |  |
|  | Living with spouse/partner  (vs. living alone) | 0.011  (-0.117, 0.139) | 0.376  (-4.033, 4.784) | 2.246 | 0.17 | 0.867 | 4.11 |  |
|  | Living with children (two generations)  (vs. living alone) | 0.050  (-0.074, 0.173) | 1.859  (-2.782, 6.501) | 2.365 | 0.79 | 0.432 | 3.86 |  |
|  | Living with three or more generations  (vs. living alone) | 0.049  (-0.051, 0.150) | 2.478  (-2.586, 7.541) | 2.580 | 0.96 | 0.337 | 2.56 |  |
|  | Fair economic status  (vs. satisfied) | -0.065  (-0.132, 0.001) | -2.265  (-4.558, 0.029) | 1.169 | -1.94 | 0.053 | 1.11 |  |
|  | Dissatisfied with economic status (vs. satisfied) | -0.048  (-0.114, 0.019) | -4.282  (-10.30, 1.734) | 3.065 | -1.40 | 0.163 | 1.12 |  |
|  | Not retired (vs. retired) | -0.080  (-0.167, 0.008) | -3.084  (-6.474, 0.306) | 1.727 | -1.79 | 0.075 | 1.93 |  |
|  | Online-dominant  (vs. offline-dominant) | -0.098  (-0.133, -0.029) | -4.753  (-8.085, -1.422) | 1.697 | -2.80 | 0.005^**^ | 1.18 |  |
|  | Hybrid online-offline  (vs. offline-dominant) | 0.138  (0.068, 0.207) | 4.999  (2.490, 7.508) | 1.278 | 3.91 | <0.001^***^ | 1.20 |  |
|  | Non-exercise-only  (vs. exercise-only) | -0.233  (-0.359. -0.106) | -8.148  (-12.57, -3.728) | 2.252 | -3.62 | <0.001^***^ | 4.02 |  |
|  | Exercise + other activities (vs. exercise-only) | -0.118  (-0.245, 0.010) | -4.068  (-8.472, 0.336) | 2.244 | -1.81 | 0.070 | 4.10 |  |
|  | ≤1 time/week  (vs. ≤1 time/month) | 0.028  (-0.041, 0.097) | 1.009  (-1.445, 3.462) | 1.250 | 0.81 | 0.420 | 1.19 |  |
|  | ≥2 times/week  (vs. ≤1 time/month) | -0.122  (-0.191, -0.053) | -5.555  (-8.679, -2.431) | 1.591 | -3.49 | 0.001^**^ | 1.19 |  |
|  | Constant | - | 83.734 | 3.054 | 27.41 | <0.001 | - |  |

^*^*p* < 0.05. ^**^*p* < 0.01. ^***^*p* < 0.001.
